# Species, Diaspore Volume and Body Mass Matter in Gastropod Seed Feeding Behavior

**DOI:** 10.1371/journal.pone.0068788

**Published:** 2013-07-03

**Authors:** Manfred Türke, Wolfgang W. Weisser

**Affiliations:** Institute of Ecology, Friedrich-Schiller-University, Jena, Germany; University of Copenhagen, Denmark

## Abstract

**Background:**

Seed dispersal of ant-dispersed plants (myrmecochores) is a well studied ecosystem function. Recently, slugs have been found to act as seed dispersers of myrmecochores. The aim of our study was to (1) further generalize the finding that gastropods feed on seeds of myrmecochores and hence may act as seed dispersers, (2) to test whether gastropod body mass and the volume of diaspores have an influence on the seed dispersal potential.

**Methodology and Principal Findings:**

We assessed the seed dispersal potential of four slug and snail species with a set of seven myrmecochorous plant species from seven different plant families common to Central European beech forests. Diaspores differed in shape and size. Gastropods differed in their readiness to feed on diaspores and in the proportion of seeds that were swallowed as a whole, and this readiness generally decreased with increasing diaspore size. Smaller Arionid slugs (58 mm body length; mean) mostly fed on the elaiosome but also swallowed small diaspores and therefore not only act as elaiosome consumers, a nutrient rich appendage on myrmecochorous diaspores, but may also disperse seeds. Large Arionid slugs (>100 mm body length) swallowed diaspores of all sizes. Diaspores swallowed by gastropods were defecated without damage. Within-species variability in body size also affect seed dispersal potential, as larger individuals of the red slug (*Arion rufus*) swallowed more diaspores of wood anemone (*Anemone nemorosa*) than smaller ones.

**Conclusions and Significance:**

Our results help to generalize the finding that gastropods consume and potentially disperse seeds of myrmecochores. The dispersal potential of gastropods is strongly influenced by diaspore size in relation to gastropod size.

## Introduction

Seed dispersal by ants (myrmecochory) was a driver of an incredible diversification within the group of angiosperms [1]. Thousands of plant species worldwide rely on ants as dispersal vectors of their diaspores and many ant-dispersed plant species, called myrmecochores, are coexisting in certain habitats [2]–[4]. Dispersal of diaspores by ants allows plants to reach microsites suitable for seedling establishment [2], [5]–[7] and helps diaspores to escape fire or predation by the rapid removal or by the burial of diaspores [8]–[10]. In a recent study by Türke et al. (2010) slugs were identified as seed dispersers of forest myrmecochores [11]. In these habitats, benefits of gastropodochory for the plant could potentially be greater dispersal distances permitted by gastropods than by ants [11], the availability of gastropods as seed dispersers where ants are rare [12] or it may reduce the subsequent risk of seed predation as diaspores swallowed and defecated by slugs were less attractive to rodents than untreated diaspores in the laboratory [11]. Furthermore, gut passage through slugs can even accelerate seed germination or increase total germination [13], [14].

With about 35,000 species, terrestrial gastropods are a very diverse group, with different foraging behavior and feeding habits [15], [16]. Slugs often consume the elaiosome, a nutrient-rich appendage on myrmecochorous diaspores, without swallowing and potentially dispersing the diaspore [11], [13], [17]. However, the large slugs *Arion rufus* and *A. ater* have recently been shown to swallow myrmecochorous diaspores as a whole under laboratory conditions and in the field [11], [13]. Gastropod-defecated seeds were, in general, undamaged and germinable and thus dispersed endozoochorously [11], [13], [14], [18], [19]. A recent study highlighted the importance of gastropods for the removal of myrmecochorous diaspores in beech forests in Germany [12], which is particularly interesting as myrmecochores were also highly abundant where ants were rare or missing, suggesting that gastropods might substitute ants as seed dispersers in certain habitats. Also in the field gastropods often left diaspores behind with their elaiosomes being detached [12]. Thus, gastropods serve different ecological roles, acting either as mutualists or antagonists for plant dispersal. The net effect of these interactions may vary among and within gastropod and plant species.

For some species it has been shown that the elaiosome has some chemical similarity to the insect hemolymph and in ants it has been demonstrated that mainly predatory species forage for myrmecochorous diaspores [20]–[22]. If this also applies to gastropods, one could suggest that gastropods feeding on invertebrates rather than strict herbivores should feed on myrmecochorous diaspores. In fact, recent results supported this hypothesis, but the number of gastropod and plant species involved in the study were too limited for generalization [11]. Honek et al. (2009) found that ten slug and snail species with different feeding habits consumed wind-dispersed diaspores of Dandelion (*Taraxacum* agg.) in the laboratory, which lack an elaiosome [19]. Consumption of diaspores without elaiosome was also recently observed for black slugs (*A. ater*) that consumed plant species with apparently unassisted dispersal modes [13]. Thus, we cannot exclude that the seed itself is somehow attractive to gastropods, but studies examining the nutritional benefit of seed or elaiosome consumption by gastropods are still missing.

To understand what makes a gastropod a diaspore or elaiosome consumer, and hence a plant dispersal antagonist in contrast to a plant disperser we have to study the underlying mechanisms. There are indications that diaspore size relative to gastropod size is important for seed dispersal by gastropods e.g. it was observed that while larger slug individuals swallowed large diaspores, smaller ones did not [11], [13], suggesting that gastropods might have morphological restrictions in large diaspore ingestion. Relative gastropod body size may also underlie within-species variability in seed dispersal [11]. Thus, gastropod body size may affect not only diaspore swallowing behavior but also elaiosome consumption. A similar pattern was found for earthworm diaspore consumption, which can swallow diaspores in all just like gastropods do. Smaller earthworm species ingested fewer diaspores than larger species and smaller diaspores were generally preferred over larger ones [23]. For *Lumbricus terrestris* a significant effect of body weight on seed ingestion was found, with larger individuals swallowing more diaspores [24]. Also for ants, diaspore size (but also elaiosome size alone or its relation to diaspore size) and ant body size influence diaspore collecting behaviour [6], [25]–[27].

In this study, we conducted feeding experiments with diaspores of seven forest myrmecochores in the laboratory, differing in size and shape, offered to four gastropod species with different feeding habits. We asked the following questions: (1) do gastropods consume diaspores of all myrmecochores?, (2) are there differences in the feeding and seed dispersal behavior among the gastropod species that depend on gastropod body size?, and (3) what is the role of diaspore size for gastropod swallowing behaviour?

## Materials and Methods

### Plant Species

Diaspores of the following myrmecochorous early-spring flowering herbs [28] from seven different plant families were chosen for experiments: (1) bear’s garlic (*Allium ursinum* L.; Alliaceae) lack a distinct elaiosome but are surrounded by a fatty seed coat. Diaspores of (2) toothwort (*Lathraea squamaria* L.; Scrophulariaceae), (3) hedge violet (*Viola reichenbachiana* Boreau; Violaceae), (4) wood anemone (*Anemone nemorosa* L.; Ranunculaceae), (5) dog’s mercury (*Mercurialis perennis* L.; Euphorbiaceae), (6) European wild ginger (*Asarum europaeum* L.; Aristolochiaceae) and (7) yellow archangel (*Lamiastrum galeobdolon* agg. L.; Lamiaceae) all bear a more or less distinct elaiosome. Diaspores differ according to shape and size [29]. These species are commonly found in beech forests [3], [4] and were chosen for experiments as we could collect adequate numbers of diaspores of them in beech dominated forests in the Hainich region south of Mühlhausen in Thuringia, Germany, at 400 to 500 m a.s.l. (coordinates 10°27′ E/51°05′ N) and in deciduous forests surrounding the city of Jena, Thuringia (11°36′ E/50°56′ N). Diaspores were collected just before ripening (April to June) and kept in a freezer at −20°C until used in the experiments to prevent desiccation of elaiosomes.

To relate gastropod feeding behavior to diaspore dimensions, diaspores were measured digitally at 12.5 times magnification with the program COMEF_Autoshape 3.0 (OEG GmbH) using a MZM1 microscope (Mikroskop Technik Rathenow), a CF11DSP camera (Kappa optronics GmbH) and a FALCON Framegrabber (IDS Imaging Development Systems GmbH). Maximum width (W), length (L) and thickness (T) of the diaspores (fruit/seed including elaiosome) of ten randomly chosen diaspores of each species were used to calculate diaspore volume (V) as W × L × T. For *A. nemorosa,* the diaspore volume was calculated as W × L × T of the achene’s body+W × L × T of the persistent style. We chose diaspore volume rather than length or surface, as this provided the best measure describing earthworm diaspore ingestion [23]. Plant species all differed significantly in diaspore volume (V_D_; ANOVA; F_6,63_ = 60.08, p<0.001), except for the pairs of *A. ursinum* and *L. galeobdolon* and *A. ursinum* and *A. europaeum*. Diaspores ranged from very small (*L. squamaria,* V_D_ = 1.8 mm^3^, mean), small (*V. reichenbachiana,* 5.8 mm^3^) or medium-sized to large (*A. nemorosa,* 16.5 mm^3^; *L. galeobdolon,* 17.2 mm^3^; *A. ursinum*, 21.4 mm^3^; *A. europaeum*, 22.1 mm^3^; *M. perennis*, 31.8 mm^3^).

### Slugs and Snails

Experiments were conducted with native gastropods abundant in beech forests, in particular, mature and juvenile red slugs *(Arion rufus* L.; length of mature individuals 10–20 cm; [16]), ash-grey slugs *(Limax cinereo-niger* Wolf; 10–30 cm) and white-lipped snails (*Cepaea hortensis* Müller; shell height 1.0–1.7 × width 1.4–2.2 cm). We also included the invasive Spanish slug (*Arion lusitanicus* Mabille; 7–14 cm) that is currently mostly found in open habitats where it is displacing the native large slugs *A. rufus* and *A. ater* [30]–[33] but has been described to enter forests as well [31]. We included *A. lusitanicus* in our study to compare its seed feeding behavior to that of *A. rufus*. All gastropods were collected in deciduous forests in the Hainich region except for *A. lusitanicus* which was collected in a garden in Hermsdorf, Thuringia, about 40 km east of Jena. All mature slugs were larger than 10 cm when extended. Smaller, juvenile *A. rufus* measuring 58±4 mm (mean±SE) were included in this study to evaluate the influence of within-species variability on slug body size on their seed feeding behaviour. Species were chosen due to their abundance in beech forests [34], their size as we supposed that all species tested could potentially disperse diaspores and because we could sample individuals of these species in adequate numbers to test their feeding behavior experimentally. Species that may also disperse diaspores but were too rarely observed to be sampled (e.g. *Helix pomatia, Arion subfuscus*) were not included in the study.

Gastropods were kept in the climate chamber at 20°C and 75% humidity. Slugs of more than 5 cm body length were kept in fauna boxes measuring 27 × 17 × 18 cm L × W × H (Savic Fauna box –6 L.; http://www.savic.be). Slugs of less than 5 cm body length and snails were kept in 9 cm petri-dishes. Gastropods were fed once a week with lettuce, carrots and wild herbs. All food was removed one day before an experiment. Gastropods were kept on wet paper towels, which most gastropods fed on frequently.

### Diaspore Feeding Experiments

#### Gastropod species and diaspore type interaction

To answer whether gastropods generally consume diaspores of myrmecochores and whether there are differences in the feeding among gastropod and plant species, we conducted diaspore feeding experiments in the laboratory. Gastropod individuals of all species were offered six diaspores of one of the seven plant species at a time except for *A. europaeum* where only five diaspores were offered to *A. rufus*. Diaspores of *L. galeobdolon* were only offered to mature and juvenile *A. rufus* as numbers were limited. Diaspores of a plant species were offered 5 to 28 individuals (14±1 mean±SE) of a species (Table 1). This variance was due to a limited number of gastropod individuals in some species. In total we used three gastropod species × 6 plant species = 18 plus additionally two age/size classes of *A. rufus* × 7 plant species = 14, in total 32 gastropod-plant combinations. Experiments were conducted from August to October, 2007.

**Table 1:** Number of gastropod individuals to which diaspores of certain plant species were offered (No) and proportion of individuals that fed on the diaspores (Nf(%)).

	*A. rufus* (mature)		*A. rufus* (juvenile)		*L. cinereo-niger*		*A. lusitanicus*		*C. hortensis*	
	No	Nf(%)	No	Nf(%)	No	Nf(%)	No	Nf(%)	No	Nf(%)
Elaisome damage not visible										
*A. ursinum*	16	38	5	0	17	0	13	38	11	0
*L. squamaria*	16	63	10	50	17	0	8	75	12	8
*V. reichenbachiana*	15	67	9	11	19	16	11	91	12	0
Elaisome damage visible										
*A. nemorosa*	21	57	13	54	19	16	15	60	13	54
*M. perennis*	21	90	10	90	18	89	9	100	13	85
*A. europaeum*	28	100	13	69	19	16	17	47	13	38
*L. galeobdolon*	19	47	9	89	–	–	–	–	–	–

In species where elaiosome damage was visible, feeding included swallowed seeds and seeds with consumed elaiosomes and in species where elaiosome damage was not visible, only swallowed seeds are regarded as fed upon.

Diaspores were exposed to gastropods for 48 hours and were checked after 24 and 48 hours. For each diaspore we distinguished between the following categories: (1) swallowing of the whole diaspore, (2) consumption of parts of or the entire elaiosome, (3) no feeding (disregarded). Some individuals were offered diaspores of several plant species and we accounted for the re-use of individuals in the analysis. In case diaspores were apparently undamaged, they were examined under the microscope at 65-times magnification. Feces were collected after 24 hours and 48 hours to search for digested and defecated diaspores. Defecated diaspores were also examined under the microscope.

Results of the feeding experiments with *A. nemorosa* and *A. europaeum* by mature and juvenile *A. rufus* and by *L. cinereo-niger* were published in Türke et al. (2010) [11]. These original data were a small part of the larger dataset presented in this manuscript. We integrated the whole data into our multi-species analysis to describe particular patterns of gastropod diaspore feeding behavior and to compare it among a number of plant and gastropod species.

#### Slug body mass and diaspore feeding behavior

As it turned out in previous experiments that there was considerable variation in the diaspore feeding behavior within each gastropod species, we designed another experiment to test for an influence of body size on the seed feeding behavior of mature *A. rufus* individuals. We collected 273 supposedly mature individuals (>10 cm body length) of *A. rufus* in beech forests in the Hainich region on May 19^th^ and 20^th^, 2009, a time at which *A. nemorosa* sheds its diaspores. All mature individuals of *A. rufus* that we discovered were collected at each forest site. The size of the area searched for slugs varied as slug abundance also varied between sites. Slugs were brought to the laboratory on May 20^th^, 2009 and the live body mass was measured with a “Monobloc inside” balance (Mettler-Toledo GmbH; accuracy 0.001 g). Mean body mass was 11.0±3.5 g (mean±SD), ranging 3.9 g to 24.0 g.

All individuals were assigned to four body mass classes: (1) the minimum to the 25% quartile of body mass of all individuals (3.9–8.6 g), (2) more than the 25% quartile to the median (8.7–10.9 g), (3) more than the median to the 75% quartile (11.0–13.2 g) and (4) more than the 75% quartile to the maximum (13.3–24.0 g). We randomly selected 14 individuals of each group for the subsequent experiment. The individuals were fed with wild herbs (mainly dandelion *Taraxacum* ssp. leaves) for three days, and were then starved for another three days (except for the wet paper in the box on which individuals fed frequently). Then we offered 10 diaspores of *A. nemorosa* and a small amount of potato (0.3 g) to each slug. Potato is a food attractive to slugs and feeding on it was used to indicate slug activity during the experiment. If no diaspores but potato were consumed we could assume that diaspores were not attractive to slugs. If neither potato nor diaspores were consumed we could exclude these inactive slugs from the analysis (which, however, was not necessary). After 48 hours we assessed the number of diaspores swallowed and for all remaining diaspores the proportion of fruit skin area that was consumed. Swallowed diaspores have 100% of their fruit skin consumed. The fruit skin area consumed per diaspore was summed over all diaspores for each individual and then we calculated the proportion of fruit skin area consumed of the total fruit skin of all ten diaspores.

### Statistical Analysis

Figures were created using SigmaPlot 11.0 (Systat Software Inc.). Statistical analysis was carried out using R 2.14.1 (R Development Core Team; http://www.r-project.org/). Diaspore volumes of plant species were transformed to obtain normality and compared with ANOVA using the function “aov”. In the gastropod feeding experiment, we compared the number of swallowed diaspores and of diaspores with consumed elaiosomes among gastropod species and plant species in a generalized linear mixed-effects model (fit by the Laplace approximation for binomial errors) using the function “glmer” in the lme4 package. Plant species and gastropod species were treated as fixed effects and gastropod individuals as a random effect. We tested for overdispersion of the models (see Appendix S1). We performed multiple comparisons of means (Tukey contrasts) using the function ‘‘glht’’ in the multcomp package. To derive global statistic measures for the mixed models we further conducted a global test using the formula: K <- diag(length(fixef(model)))[ −1,] and summary(glht(model, K = K), test = Chisqtest()). The number of swallowed diaspores was further analyzed in a GLMM for its dependency on diaspore volume (fixed effect) with gastropod individual as a random effect.

For the slug body mass and diaspore feeding behavior experiment, we performed a Fisher’s exact test to compare the number of individuals of the four body mass classes that swallowed diaspores using the function “fisher.test”. We further tested for a correlation (Spearman’s Rank Correlation with the function “cor.test” and the method = ”spearman”) of slug body mass with (1) the number of diaspores swallowed, (2) the proportion of total fruit skin area consumed of all ten diaspores and (3) with the proportion of fruit skin consumed by swallowing diaspores of the total fruit skin area consumed of all ten diaspores.

## Results

### Gastropod Species and Dispersal Potential

**Figure 1:**
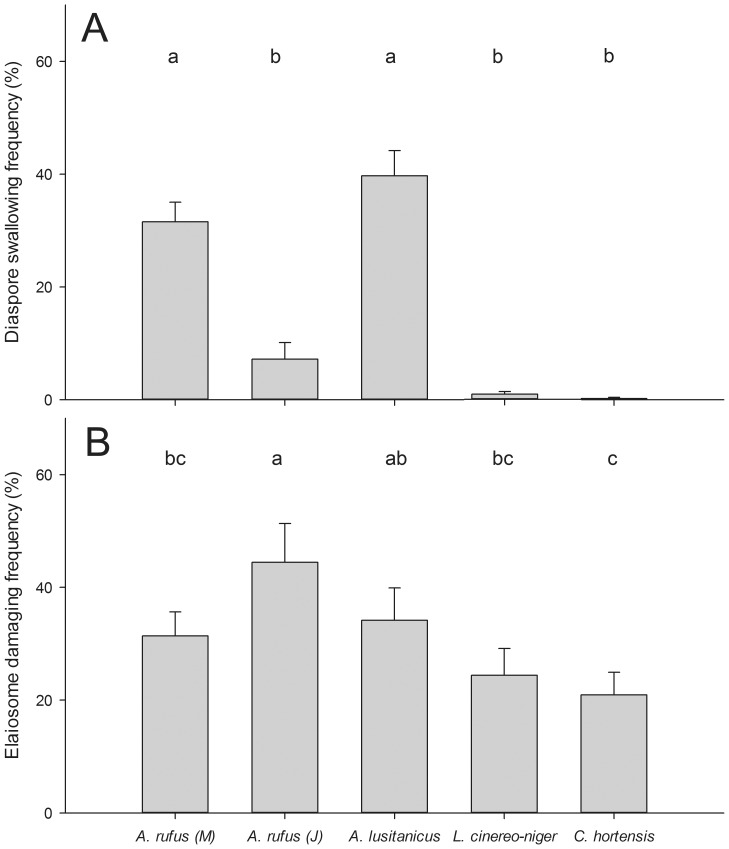
Comparison of diaspore swallowing and the elaiosome damaging frequency among gastropods. Comparison of (A) the diaspore swallowing frequency and (B) of the elaiosome damaging frequency among gastropod species. Swallowing of diaspores is considered as the potential dispersal ability of gastropods. Lamiastrum galeobdolon was not offered to all gastropod species and is not shown here. M, mature individuals; J, juveniles. Results are given as mean ± standard error (SE). Results of GLMM analyses (Tukey contrasts) are shown for comparisons of diaspore and elaiosome feeding; treatments with different letters differ significantly at least at p<0.05.

**Figure 2:**
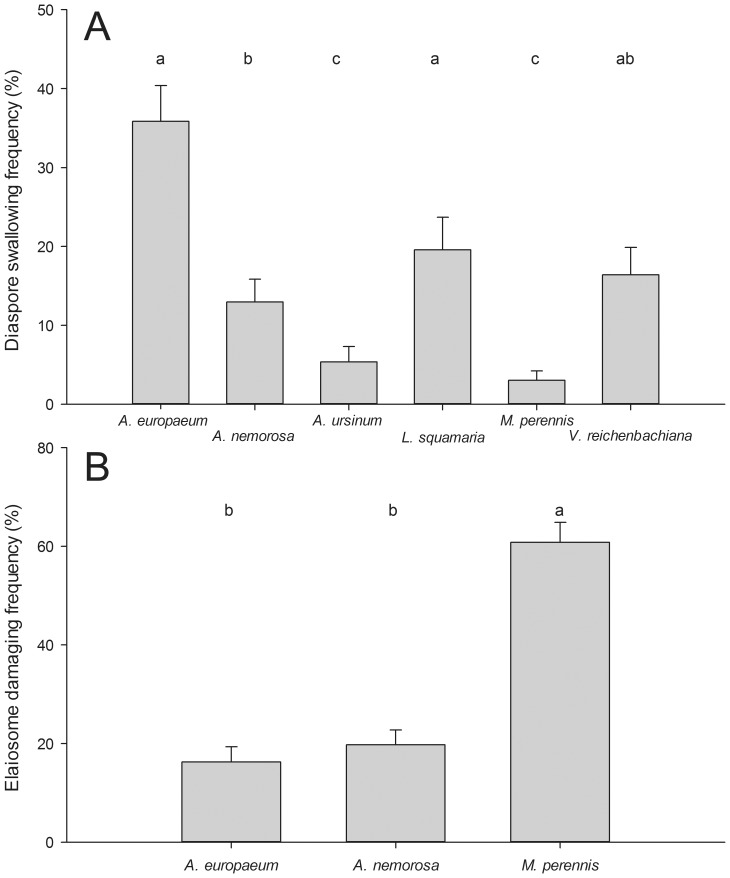
Comparison of diaspore swallowing and the elaiosome damaging frequency by gastropods among plant species. Comparison of (A) the diaspore swallowing frequency and (B) of the elaiosome damaging frequency by gastropod species among plant species. Swallowing of diaspores is considered as the potential dispersal ability of gastropods. Lamiastrum galeobdolon was not offered to all gastropod species and is not shown here. Results are given as mean ± standard error (SE). Results of GLMM analyses (Tukey contrasts) are shown for comparisons of diaspore and elaiosome feeding; treatments with different letters differ significantly at least at p<0.05.

**Figure 3:**
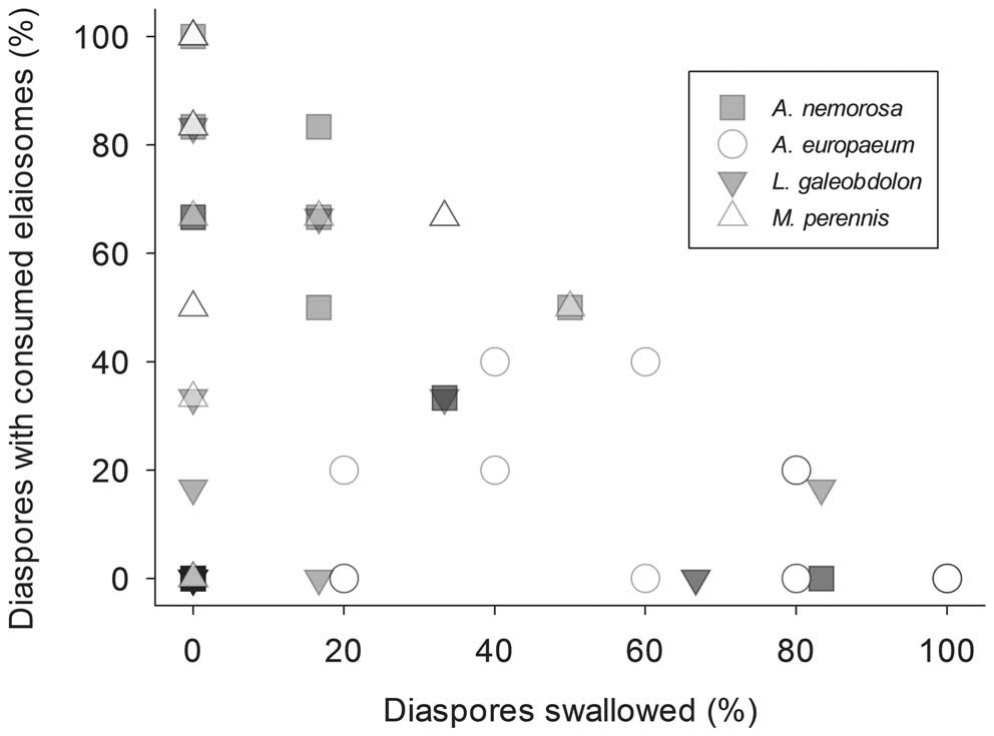
Different feeding behavior of diaspores by *A. rufus individuals.* Relationship of diaspores that were either swallowed or of which elaiosomes were consumed by *A. rufus* individuals. Each point represents a single feeding trial (N = 89). The sum of both axes may not exceed 100% ( = all diaspores offered were consumed) but may well be less if diaspores remained disregarded by slugs. There are great differences in the feeding behavior among individuals.

All gastropod species were found to feed on diaspores of at least some plant species (Fig. 1; for species pairs see Figs. S1 and S2 in the Online Appendix) and diaspores of all plant species were consumed (Fig. 2), but often only some of the gastropod individuals showed feeding behavior (Table 1). There were differences in the way diaspores of different plant species were consumed: in *A. ursinum, L. squamaria* and *V. reichenbachiana* elaiosomes were generally undamaged and no elaiosome removal or feeding traces on elaiosomes were noticed, although feeding cannot be entirely excluded as slugs and snails appeared to handle diaspores. For these plant species, therefore, only the proportions of diaspores swallowed were analysed. For diaspores of *A. nemorosa, M. perennis, A. europaeum* and *L. galeobdolon* feeding on the elaiosomes was observed and both, elaiosome feeding and swallowing of entire diaspores, was used in the statistical analysis. There were great differences among individuals within gastropod species in 1) the number of diaspores swallowed 2) the number of diaspores where the elaiosome was consumed and 3) the number of diaspores not touched (for *A. rufus* and those plant species where elaiosome damage was visible see Fig. 3). Linear mixed-effects models revealed significant differences in numbers of swallowed diaspores (N = 487 feeding trials involving N = 156 gastropod individuals; global test on the GLMM: ×^2^ = 258.3, df = 11, p<0.001; for details of the GLMM see Appendix S1) and in numbers of diaspores with consumed elaiosomes (N = 287 feeding trials involving N = 138 gastropod individuals; global test on the GLMM: ×^2^ = 322.9, df = 8, p<0.001; for details see Appendix S1) between plant species and between gastropod species (for general differences among gastropod species see Fig. 1 and among plant species see Fig. 2; for detailed information on the feeding of each plant species by certain gastropod species see Figs. S1 and S2).

Swallowed diaspores that were defecated by snails or slugs were all intact. Except for *M. perennis* and *A. nemorosa,* elaiosomes were also in most cases apparently undamaged after gut passage. In *A. nemorosa* diaspores, not only the basal part of the peduncle (which should denote the elaiosome [35]) was consumed but also parts of or the entire fruit skin of the achene. We considered all of this feeding in *A. nemorosa* as elaiosome damage. Although *V. reichenbachiana* and *L. squamaria* have distinct elaiosomes we could not observe that those were detached by gastropods, but only whole diaspores were swallowed, possibly indicating difficulties in handling these small diaspores or elaiosomes.

### Diaspore Volume and Dispersal Potential

**Figure 4:**
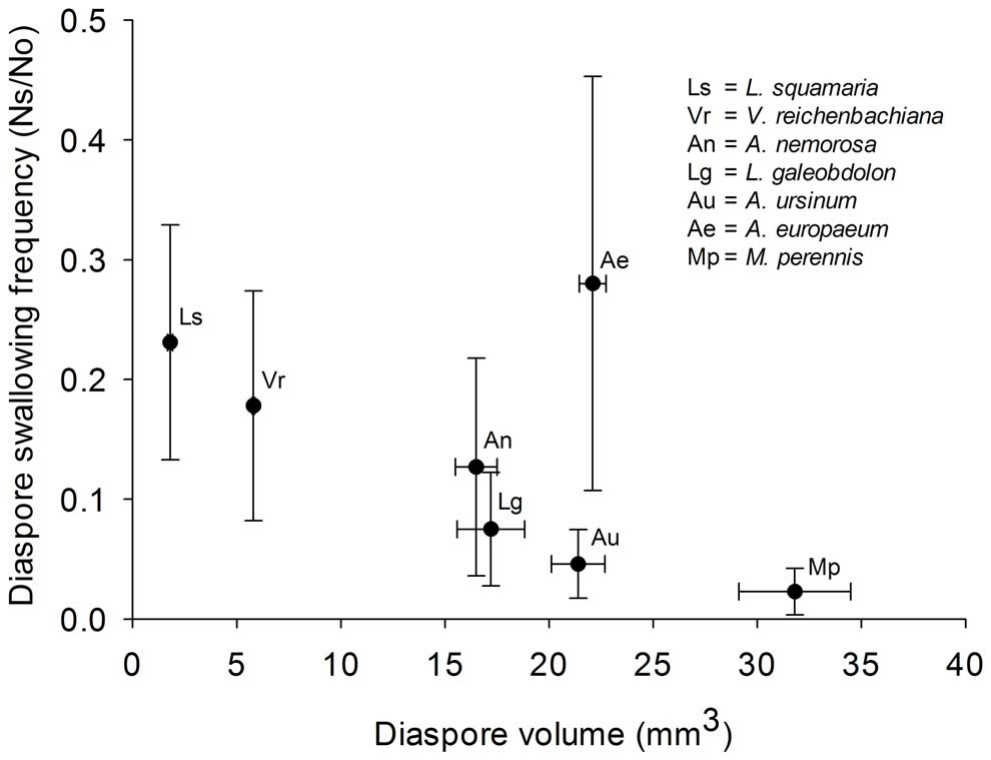
Relationship of diaspore volume and the dispersal potential. Relationship between diaspore volume and the dispersal potential of diaspores by gastropods described as the number of diaspores swallowed (Ns) of the number of diaspores offered (No) to all gastropod individuals. Results are given as mean ± standard error (SE).

Diaspore volume was negatively correlated with the proportion of diaspores swallowed by gastropods (all species combined, GLMM; z = −8.18, p<0.001; Fig. 4; for details of the GLMM see Appendix S1). *Asarum europaeum* was an exception. Its diaspores are among the largest in the set but the large Arionid slugs swallowed a much higher proportion of them than of the other diaspores offered.

### Intraspecific Slug Body Mass Variation and Dispersal Potential

**Figure 5:**
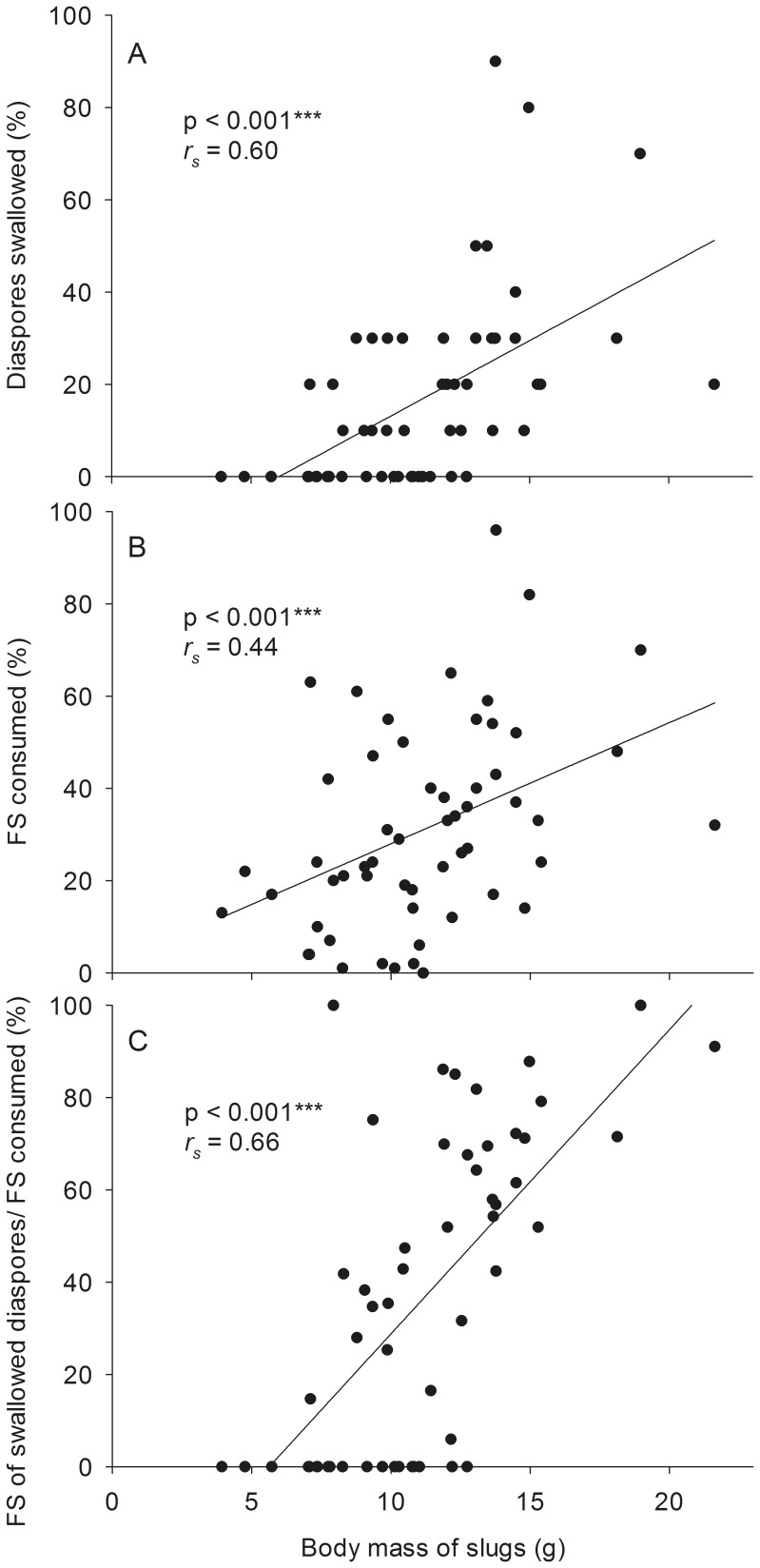
Relationship of slug body mass and diaspore swallowing frequency. Relationship of slug body mass and (A) the proportion of diaspores swallowed, (B) the proportion of diaspore fruit skin area (FS) consumed (a swallowed diaspore was regarded as if 100% of its fruit skin was consumed) and (C) the proportion of fruit skin (FS) that was consumed by swallowing diaspores of the total fruit skin area consumed of all ten diaspores. Least-squares regression lines are given to illustrate the trends. rs, Spearman’s Rank Correlation Coefficient; p, p-value of the correlation.

*Arion rufus* individuals swallowed 1.7±0.3 diaspores (mean±SE) of *A. nemorosa* and consumed 31±3% of the total fruit skin area of all ten diaspores (including swallowed diaspores; mean±SE). Just a single individual did not consume fruit skin of any diaspore. The proportion of slug individuals that swallowed diaspores increased with increasing body mass (Fisher’s exact test; p<0.001; Table 2). Larger slugs also swallowed significantly more diaspores than smaller individuals (Spearman’s Rank Correlation; N = 56, *r_s_* = 0.60, p<0.001; Fig. 5 A; Table 2), consumed more fruit skin (N = 56, *r_s_* = 0.44, p<0.001; Fig. 5 B; Table 2) and consumed a higher proportion of fruit skin through swallowing of the diaspore rather than by rasping it (N = 55, *r_s_* = 0.66, p<0.001; Fig. 5 C).

**Table 2:** Number of swallowed diaspores (mean±SE) and proportion of total fruit skin area consumed of all 10 diaspores (mean±SE) of *A. nemorosa* offered to *A. rufus* individuals within the four body mass classes (N = 14 individuals/class).

	Body mass classes			
	Class 1	Class 2	Class 3	Class 4
Range of body mass (g)	3.9–8.6	8.7–10.9	11.0–13.2	13.3–24.0
Individuals that swallowedseeds	4	7	9	14
Number of seeds swallowed	0.4±0.2	1.1±0.4	1.5±0.4	3.8±0.7
Fruit skin area consumed	19±4	27±5	31±5	47±6

## Discussion

In previous studies, gastropods have been shown to consume, defecate and disperse viable seeds, including those of some myrmecochores [11], [13], [14], [18], [19], [36]. Our study was intended to test the hypothesis that gastropods in beech forests are generally able to swallow diaspores of myrmecochores such that they may serve as seed dispersers of these plants. The results from our no-choice feeding experiments involving seven plant and four gastropod species mostly support this hypothesis as all gastropod species not only fed on diaspores but also swallowed at least some of the diaspores intact. Some gastropod species, however, only rarely swallowed diaspores of a few plant species only. We also found that the size of diaspores and the size of the gastropods strongly influence diaspore feeding behaviour and consequently the dispersal probability. This is discussed below.

Previous studies on seed dispersal by gastropods investigated fleshy fruits [14], [18], [36], plants with wind-dispersed diaspores [19], with an unassisted dispersal mechanism [13], [37] and some ant-dispersed diaspores [11]–[13]. Gastropods have a limited range for seed dispersal within a few meters [11] and thus are presumably most relevant as dispersers for myrmecochores and plants with unassisted seed dispersal as diaspores transported by wind or vertebrates are dispersed over greater distances. As diaspores with an unassisted dispersal mode lack any animal attractant and are defecated by slugs without damage [13], the reasons why gastropods consume them is up to speculations. In myrmecochores, gastropods may also consume the elaiosome exclusively as demonstrated in our study, indicating that the elaiosome is attractive to gastropods rather than or in addition to the seed itself. Future studies should test if the seed itself is attractive to gastropods by offering seeds with detached elaiosomes. As consumption of the elaiosome without ingestion of the seed will not lead to its dispersal but also prevent dispersal by ants [6], it is crucial to understand which mechanisms influence this “decision” in the feeding behavior of gastropods. It has to be announced, however, that removal of elaiosomes by gastropods can also have positive effects on seed germination [13].

### The Dispersal Potential of Gastropod Species

While all gastropod species swallowed at least some diaspores of some plant species, there were large differences among the gastropod-plant pairings. In *L. cinereo-niger* and *C. hortensis* few individuals swallowed diaspores and if they did, they swallowed only a small number of them. Thus, their importance as seed dispersers is probably low. However, both consumed elaiosomes of *M. perennis* regularly, indicating that these gastropods may well act as dispersers for plant species not tested in this study. In contrast, large Arionid slugs swallowed diaspores of all plant species offered and thus bear the potential to be significant seed dispersers, especially as they are highly abundant in many habitats [16].

We included invasive *A. lusitanicus* in our study to compare its diaspore feeding behavior to that of *A. rufus*, which is currently outcompeted in several of its native habitats [30], [32]. Both slugs were very similar in the proportions of diaspores they swallowed of each species or the number of diaspores of which elaiosomes were consumed. However, large seeded plants might suffer from the loss of the native slug as A. *lusitanicus* is smaller (up to 15 cm) than *A. rufus* (sometimes exceeding 20 cm). We should furthermore consider that both species differ in other means of behavior, which might impact on seed dispersal as well. But more studies comparing the movement and diaspore feeding behavior of both species are needed.

### Diaspore and Gastropod Size

Smaller diaspores were generally more frequently swallowed than larger diaspores. We assume that there are morphological restrictions preventing small gastropods swallowing large diaspores which partly describe the pattern we found. This was also suggested in other studies with slugs [11], [13]. As food items such as leaves are normally rasped by the radula of a gastropod, studies relating the size of food items to gastropod size would be helpful but we found none. Earthworms, however, ingesting diaspores similarly to gastropods showed a clear preference for small diaspores as well [23], [38]. Interestingly, also ants are known to face morphological restrictions in the size of diaspores they can carry in their mandibles and smaller ants transport diaspores over shorter distances [6], [26]. It has also to be announced that elaiosome size alone or its relation to diaspore size influence seed collecting behavior by ants [25] and this might apply to gastropods as well. To test these assumptions diaspores of the same plant species with high variation in seed and elaiosome size should be offered to gastropods [25]. Seed preferences by ants are also influenced by the chemical content of elaiosomes and seeds [27]. Experiments relating diaspore chemistry to gastropod seed feeding are still missing.

### Within-species Variability

Our feeding experiment with different-sized *A. rufus* individuals showed that (1) more large slugs swallowed diaspores than smaller slugs and (2) slugs with greater body mass swallowed more diaspores than smaller individuals (also in relation to the total fruit skin area consumed of all ten diaspores). Thus, large slugs are more effective seed dispersers of diaspores of *A. nemorosa* than small individuals, which will mainly act as elaiosome consumers by rasping the fruit skin of the achene without moving it. Again, the same result was found for the earthworm *Lumbricus terrestris*, with larger individuals ingesting more diaspores than smaller ones [24]. Eisenhauer et al. (2010) do not provide an explanation why larger earthworms swallow more diaspores, as also small individuals appeared not to be saturated in the experiment [24] [Eisenhauer, pers. comm.]. We can also only speculate why slugs do not ingest all diaspores, but consuming the elaiosome of some or all.

Interestingly, there is a high variability of body mass within individuals of the same age of the leopard slug (*Limax maximus* L.) and the banana slug (*Ariolimax columbianus* Gould*)* in natural habitats [39], [40]. We found that there were significant differences in body mass among individuals of *A. rufus* sampled in different forests in the same region (results not shown). In this way, habitat parameters might impact on slug body size and consequently probably also on the seed dispersal potential of the whole slug population. In our study, however, we have to concede that slugs were not sampled in a standardized way which would be necessary to compare the body mass distribution of gastropod populations and the possible consequences for seed dispersal. The density of gastropods, of course, will have a strong impact on the seed dispersal potential of the population, too.

### The Shift from Exploitation to Mutualistic Interactions

Gastropods large enough to swallow and consequently disperse diaspores of a plant species (reward for the plant) make a 'decision' whether to swallow a diaspore or to consume the elaiosome instead which will prevent dispersal by this individual but also by other gastropods or ants (no reward). Bronstein (2001) [41] called individuals with alternative behaviors of either offering commodities in return for being rewarded or failing to do so as *conditional exploiters* of mutualisms. Ants are known to act similarly. They may either pick up a diaspore and carry it to their nest providing dispersal or they consume the elaiosome on the spot without moving the diaspore. It was proposed that competition with other ants might have an influence on this decision [6]. For gastropods the same reason might apply as swallowing a diaspore takes much less time than consuming the elaiosome (pers. observations). Thus, swallowing diaspores could be an option to ace out competitors ingesting as many diaspores as possible within a short time. As gastropods were kept singly in fauna boxes in our experiment, however, competition appears not to be a good or the only explanation for the conditional exploitation behavior we observed and other factors must influence the feeding behavior as well.

As body size of gastropods restricts the capability of swallowing diaspores of a certain size or plant species, some gastropod species will always act as exploiters for certain plants as they are just too small to swallow diaspores of these species. These gastropods could be seen as *pure exploiters* of the mutualistic plants [41]. The same observation of size restriction in seed dispersal was made for ants, with small ants providing shorter dispersal distances or not moving diaspores at all [26]. More interesting, however, is the fact that gastropods have very similar food preferences throughout their ontogeny and that they increase incredibly in size during their lifetime. Our results suggest that at a certain threshold of body size, gastropods will start swallowing and dispersing diaspores that they would not swallow when smaller. Thus, during its ontogeny a gastropod will switch from being a pure exploiter to a conditional exploiter or a mutualist for the same plant species. Results further suggest that by increasing in size the relation of exploitation (elaiosome consumption) to mutualistic behavior (swallowing diaspores) is constantly decreasing. We are not aware of any mutualistic partnership where there is a comparable ontogenetic shift from exploiter to mutualist within the same species. Some similarity can be found in the yucca/yucca moth mutualism where female adult moths pollinate the yucca plant (reward for the plant) and the larvae of the same female predate on the seeds of the plant (reward for the moth) [42]. Moth larvae, however, consume only a small part of the seeds produced by the plant and it is the same plant individual that will receive the reward of pollination and that 'offers' seeds as commodity in return. The seeds consumed can be seen as an analogue to a food body or elaiosome. The differences are that in the case of the gastropod exploitation of the mutualistic partner, different plant individuals will be visited by a gastropod individual when it is juvenile (non-seed dispersing) and when it becomes adult (seed dispersing). Furthermore, all diaspores (respectively their elaiosomes) that will be consumed by a juvenile will not be dispersed and this could happen to all the diaspores produced by one plant individual and not just to a part of them.

### Conclusion

Our study has confirmed that gastropods feed on diaspores of myrmecochores and are capable of dispersing a variety of diaspores differing in size and shape. Gastropods may exhibit different functional traits for plants, either beneficial or detrimental, when dispersing diaspores or consuming elaiosomes. Smaller seeded plants might benefit most from gastropodochory concerning the proportion of seeds dispersed. The involvement of gastropods in seed ecology of myrmecochores makes this diffuse mutualism even more complex, but nevertheless we strongly encourage researchers to account for the seed dispersal by gastropods in their studies.

## Supporting Information

Figure S1 **Diaspores of plants where elaiosome damage was visible consumed by gastropods.** Fate of diaspores of myrmecochorous plants where elaiosome damage was visible offered to slugs and snails. Diaspores were either swallowed (open bars) or had their elaiosomes damaged or removed by feeding (grey bars). Results are given as mean ± SE.
(TIF)

Figure S2 **Diaspores of plants where elaiosome damage was not visible swallowed by gastropods.** Proportion of diaspores of myrmecochorous plants where elaiosome damage was not visible swallowed by slugs and snails. Elaiosome feeding – though not visible on diaspores – could not totally be excluded as we observed gastropods handling diaspores without swallowing them. The actual feeding on the diaspores might thus be underestimated. Results are given as mean ± SE.
(TIF)

Appendix S1 **Detailed results of mixed models in the gastropod feeding experiment.** Detailed results of the generalized linear mixed-effects models and Tukey post-hoc tests for differences in the number of swallowed diaspores and of diaspores with consumed elaiosomes among gastropod species and plant species in the gastropod feeding experiment.
(PDF)
