# Supplementary figures and images for: Species, Diaspore Volume and Body Mass Matter in Gastropod Seed Feeding Behavior
Source: PLoS One. 2013 Jul 3;8(7):e68788. doi: 10.1371/journal.pone.0068788 (PMC3700971; doi:10.1371/journal.pone.0068788)

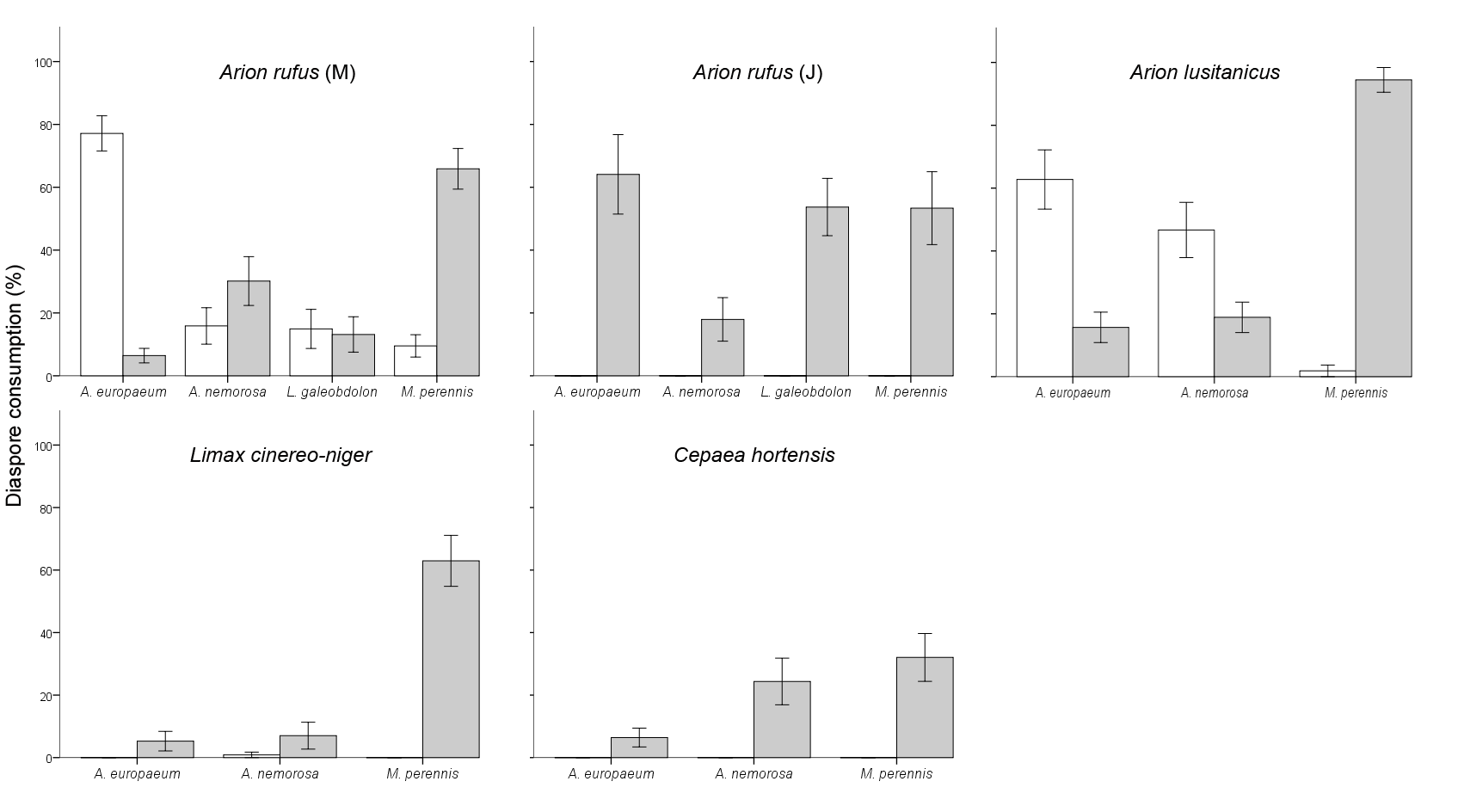

Supplement: Figure S1 — Diaspores of plants where elaiosome damage was visible consumed by gastropods. Fate of diaspores of myrmecochorous plants where elaiosome damage was visible offered to slugs and snails. Diaspores were either swallowed (open bars) or had their elaiosomes damaged or removed by feeding (grey bars). Results are given as mean ± SE. (TIF) [file pone.0068788.s001.tif]

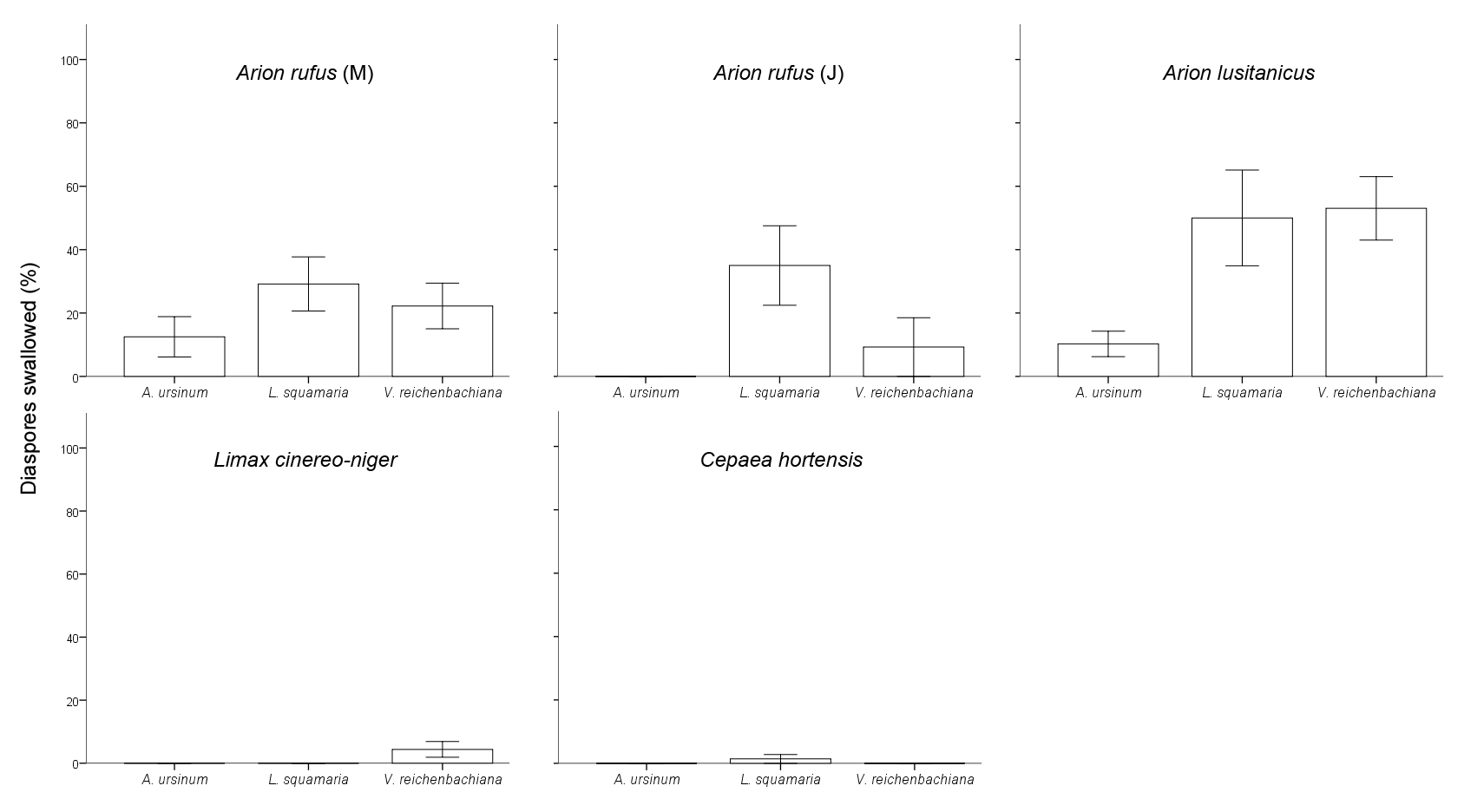

Supplement: Figure S2 — Diaspores of plants where elaiosome damage was not visible swallowed by gastropods. Proportion of diaspores of myrmecochorous plants where elaiosome damage was not visible swallowed by slugs and snails. Elaiosome feeding – though not visible on diaspores – could not totally be excluded as we observed gastropods handling diaspores without swallowing them. The actual feeding on the diaspores might thus be underestimated. Results are given as mean ± SE. (TIF) [file pone.0068788.s002.tif]
