# Supplementary material for: Species, Diaspore Volume and Body Mass Matter in Gastropod Seed Feeding Behavior
Source: PLoS One. 2013 Jul 3;8(7):e68788. doi: 10.1371/journal.pone.0068788 (PMC3700971; doi:10.1371/journal.pone.0068788)
Supplement: Appendix S1 — Detailed results of mixed models in the gastropod feeding experiment. Detailed results of the generalized linear mixed-effects models and Tukey post-hoc tests for differences in the number of swallowed diaspores and of diaspores with consumed elaiosomes among gastropod species and plant species in the gastropod feeding experiment. (PDF) [file pone.0068788.s003.pdf]

Appendix S1: Statistical summary

1.) Test for overdispersion:

We tested whether residual deviance/residual degrees of freedom are about 1 using the formula:

```
> dispersion_glmer <- function(modelglmer)
+ {
+ n <- length(modelglmer@resid)
+ return( sqrt( sum(c(modelglmer@resid, modelglmer@u) ^2) / n ) )
+ }
> model<-glmer(y~factor)
> dispersion_glmer(model)
```

2.1) GLMM for differences in the number of swallowed seeds (swallowbind) among gastropod (slug_species) and plant species (plant) with gastropod individuals as random factor (slug_ID):

```
> gastropod<-glmer(swallowbind~plant+slug_species+(1|slug_ID),
data=dataset1, family=binomial)

> dispersion_glmer(gastropod)
[1] 1.121061

> summary(gastropod)

Generalized linear mixed model fit by the Laplace approximation
Formula: swallowbind ~ plant + slug_species + (1 | slug_ID)
Data: dataset1
AIC BIC logLik deviance
756.1 805.7 -366 732.1
Random effects:
Groups Name Variance Std.Dev.
slug_ID (Intercept) 2.5796 1.6061
Number of obs: 461, groups: slug_ID, 151
Fixed effects:
Estimate Std. Error z value Pr(>|z|)
(Intercept) -2.1800 0.5046 -4.320 1.56e-05 ***
plantAnemone 1.5670 0.3522 4.449 8.61e-06 ***
plantAsarum 2.8662 0.3518 8.148 3.71e-16 ***
plantLamium 1.2034 0.4561 2.638 0.00833 **
plantLathrea 2.8271 0.3654 7.738 1.01e-14 ***
plantMercurialis -0.1609 0.4525 -0.356 0.72218
plantViola 2.2302 0.3502 6.367 1.92e-10 ***
slug_speciesArufjuvenil -3.5140 0.6939 -5.064 4.11e-07 ***
slug_speciesArufus -0.6513 0.4653 -1.400 0.16154
slug_speciesCepaea -7.0552 1.5763 -4.476 7.62e-06 ***
slug_speciesLcinereo -5.7341 0.8256 -6.945 3.78e-12 ***
---
Signif. codes: 0 ‘***’ 0.001 ‘**’ 0.01 ‘*’ 0.05 ‘.’ 0.1 ‘ ’ 1
Correlation of Fixed Effects:
(Intr) plntAn plntAs plntLm plntLt plntMr plntVl slg_spcsArfj
plantAnemon -0.511
plantAsarum -0.531 0.728
plantLamium -0.353 0.547 0.545
plantLathre -0.469 0.670 0.707 0.528
plantMrcrls -0.381 0.553 0.556 0.469 0.552
```

```
plantViola -0.487 0.697 0.716 0.556 0.689 0.542
slg_spcsArfj -0.474 -0.003 -0.030 -0.042 -0.053 -0.011 -0.025
slg_spcsArfs -0.726 0.007 -0.006 -0.073 -0.028 -0.024 -0.006 0.536
slug_spcsCp -0.209 0.002 -0.011 -0.007 -0.027 -0.003 -0.012 0.159
slg_spcsLcn -0.399 0.002 -0.023 -0.014 -0.045 -0.006 -0.025 0.304
slg_spcsArfs slg_sC
R Console Page 34
plantAnemon
plantAsarum
plantLamium
plantLathre
plantMrcrls
plantViola
slg_spcsArfj
slg_spcsArfs
slug_spcsCp 0.235
slg_spcsLcn 0.448 0.134
```

2.2) Tukey post-hoc test for significant differences between plant species:

```
> gastropod1<-glht(gastropod, lin=mcp(plant='Tukey'))

> summary(gastropod1)

Simultaneous Tests for General Linear Hypotheses
Multiple Comparisons of Means: Tukey Contrasts
Fit: glmer(formula = swallowbind ~ plant + slug_species + (1 | slug_ID),
data = dataset1, family = binomial)
Linear Hypotheses:
Estimate Std. Error z value Pr(>|z|)
Anemone - Allium == 0 1.56703 0.35219 4.449 < 0.001 ***
Asarum - Allium == 0 2.86616 0.35178 8.148 < 0.001 ***
Lamium - Allium == 0 1.20339 0.45613 2.638 0.10890
Lathrea - Allium == 0 2.82711 0.36535 7.738 < 0.001 ***
Mercurialis - Allium == 0 -0.16090 0.45254 -0.356 0.99983
Viola - Allium == 0 2.23016 0.35024 6.367 < 0.001 ***
Asarum - Anemone == 0 1.29913 0.25940 5.008 < 0.001 ***
Lamium - Anemone == 0 -0.36364 0.39552 -0.919 0.96777
Lathrea - Anemone == 0 1.26008 0.29171 4.320 < 0.001 ***
Mercurialis - Anemone == 0 -1.72793 0.39056 -4.424 < 0.001 ***
Viola - Anemone == 0 0.66313 0.27345 2.425 0.17960
Lamium - Asarum == 0 -1.66277 0.39596 -4.199 < 0.001 ***
Lathrea - Asarum == 0 -0.03905 0.27486 -0.142 1.00000
Mercurialis - Asarum == 0 -3.02706 0.38914 -7.779 < 0.001 ***
Viola - Asarum == 0 -0.63601 0.26443 -2.405 0.18698
Lathrea - Lamium == 0 1.62372 0.40696 3.990 0.00118 **
Mercurialis - Lamium == 0 -1.36429 0.46842 -2.913 0.05230 .
Viola - Lamium == 0 1.02677 0.39112 2.625 0.11241
Mercurialis - Lathrea == 0 -2.98801 0.39482 -7.568 < 0.001 ***
Viola - Lathrea == 0 -0.59695 0.28261 -2.112 0.33305
Viola - Mercurialis == 0 2.39106 0.39433 6.064 < 0.001 ***
---
Signif. codes: 0 ‘***’ 0.001 ‘**’ 0.01 ‘*’ 0.05 ‘.’ 0.1 ‘ ’ 1
(Adjusted p values reported -- single-step method)
>
```

2.3) Tukey post-hoc test for significant differences between gastropod species:

```
> gastropod2<-glht(gastropod, lin=mcp(slug_species='Tukey'))
```

```
> summary(gastropod2)

Simultaneous Tests for General Linear Hypotheses
Multiple Comparisons of Means: Tukey Contrasts
Fit: glmer(formula = swallowbind ~ plant + slug_species + (1 | slug_ID),
data = dataset1, family = binomial)
Linear Hypotheses:
Estimate Std. Error z value Pr(>|z|)
Arufjuvenil - Alusit == 0 -3.5140 0.6939 -5.064 <0.001 ***
Arufus - Alusit == 0 -0.6513 0.4653 -1.400 0.5942
Cepaea - Alusit == 0 -7.0552 1.5763 -4.476 <0.001 ***
Lcinereo - Alusit == 0 -5.7341 0.8256 -6.945 <0.001 ***
R Console Page 35
Arufus - Arufjuvenil == 0 2.8627 0.5932 4.826 <0.001 ***
Cepaea - Arufjuvenil == 0 -3.5412 1.6180 -2.189 0.1612
Lcinereo - Arufjuvenil == 0 -2.2201 0.9030 -2.459 0.0859 .
Cepaea - Arufus == 0 -6.4038 1.5351 -4.172 <0.001 ***
Lcinereo - Arufus == 0 -5.0828 0.7441 -6.831 <0.001 ***
Lcinereo - Cepaea == 0 1.3211 1.6785 0.787 0.9247
---
Signif. codes: 0 ‘***’ 0.001 ‘**’ 0.01 ‘*’ 0.05 ‘.’ 0.1 ‘ ’ 1
(Adjusted p values reported -- single-step method)
```

3.1) GLMM for differences in the number of seeds with consumed elaiosomes (elaiobind) among gastropod (slug_species) and plant species (plant) with gastropod individuals as random factor (slug_ID):

```
> gastropod_elaio<-glmer(elaiobind~plant+slug_species+(1|slug_ID),
data=dataset1, family=binomial)

> dispersion_glmer(gastropod_elaio)
[1] 1.452421

> summary(gastropod_elaio)

Generalized linear mixed model fit by the Laplace approximation
Formula: elaiobind ~ plant + slug_species + (1 | slug_ID)
Data: dataset1
AIC BIC logLik deviance
767.2 799.6 -374.6 749.2
Random effects:
Groups Name Variance Std.Dev.
slug_ID (Intercept) 1.0364 1.0181
Number of obs: 270, groups: slug_ID, 133
Fixed effects:
Estimate Std. Error z value Pr(>|z|)
(Intercept) -1.357838 0.315638 -4.302 1.69e-05 ***
plantAsarum -0.006376 0.190507 -0.033 0.97330
plantLamium 0.401514 0.257758 1.558 0.11930
plantMercurialis 2.523758 0.187399 13.467 < 2e-16 ***
slug_speciesArufjuvenil 0.620759 0.436450 1.422 0.15494
slug_speciesArufus -0.705410 0.351320 -2.008 0.04466 *
slug_speciesCepaea -1.371039 0.458440 -2.991 0.00278 **
slug_speciesLcinereo -1.070703 0.411643 -2.601 0.00929 **
---
Signif. codes: 0 ‘***’ 0.001 ‘**’ 0.01 ‘*’ 0.05 ‘.’ 0.1 ‘ ’ 1
Correlation of Fixed Effects:
(Intr) plntAs plntLm plntMr slg_spcsArfj slg_spcsArfs slg_sC
plantAsarum -0.323
plantLamium -0.133 0.329
```

```
plantMrcrls -0.252 0.484 0.374
slg_spcsArfj -0.644 0.022 -0.097 -0.004
slg_spcsArfs -0.793 0.022 -0.121 -0.052 0.589
slug_spcsCp -0.599 0.021 -0.030 -0.122 0.443 0.558
slg_spcsLcn -0.670 0.023 -0.026 -0.109 0.493 0.619 0.488
```

3.2) Tukey post-hoc test for significant differences between plant species:

```
> gastropod_elaio1<-glht(gastropod_elaio, lin=mcp(plant='Tukey'))

> summary(gastropod_elaio1)

Simultaneous Tests for General Linear Hypotheses
Multiple Comparisons of Means: Tukey Contrasts
Fit: glmer(formula = elaiobind ~ plant + slug_species + (1 | slug_ID),
data = dataset1, family = binomial)
Linear Hypotheses:
Estimate Std. Error z value Pr(>|z|)
Asarum - Anemone == 0 -0.006376 0.190507 -0.033 1.000
Lamium - Anemone == 0 0.401514 0.257758 1.558 0.397
Mercurialis - Anemone == 0 2.523758 0.187399 13.467 <1e-04 ***
Lamium - Asarum == 0 0.407889 0.265351 1.537 0.410
Mercurialis - Asarum == 0 2.530134 0.192041 13.175 <1e-04 ***
Mercurialis - Lamium == 0 2.122244 0.255848 8.295 <1e-04 ***
---
Signif. codes: 0 ‘***’ 0.001 ‘**’ 0.01 ‘*’ 0.05 ‘.’ 0.1 ‘ ’ 1
(Adjusted p values reported -- single-step method)
```

3.3) Tukey post-hoc test for significant differences between gastropod species:

```
> gastropod_elaio2<-glht(gastropod_elaio, lin=mcp(slug_species='Tukey'))

> summary(gastropod_elaio2)

Simultaneous Tests for General Linear Hypotheses
Multiple Comparisons of Means: Tukey Contrasts
Fit: glmer(formula = elaiobind ~ plant + slug_species + (1 | slug_ID),
data = dataset1, family = binomial)
Linear Hypotheses:
Estimate Std. Error z value Pr(>|z|)
Arufjuvenil - Alusit == 0 0.6208 0.4365 1.422 0.60659
Arufus - Alusit == 0 -0.7054 0.3513 -2.008 0.25658
Cepaea - Alusit == 0 -1.3710 0.4584 -2.991 0.02271 *
Lcinereo - Alusit == 0 -1.0707 0.4116 -2.601 0.06833 .
Arufus - Arufjuvenil == 0 -1.3262 0.3651 -3.633 0.00258 **
Cepaea - Arufjuvenil == 0 -1.9918 0.4727 -4.214 < 0.001 ***
Lcinereo - Arufjuvenil == 0 -1.6915 0.4276 -3.955 < 0.001 ***
Cepaea - Arufus == 0 -0.6656 0.3923 -1.697 0.42903
Lcinereo - Arufus == 0 -0.3653 0.3374 -1.083 0.81124
Lcinereo - Cepaea == 0 0.3003 0.4422 0.679 0.95982
---
Signif. codes: 0 ‘***’ 0.001 ‘**’ 0.01 ‘*’ 0.05 ‘.’ 0.1 ‘ ’ 1
(Adjusted p values reported -- single-step method)
```

4) GLMM for the relation of the number of swallowed seeds and diaspore volume (seedsize) with gastropod individuals as random factor (slug_ID):

```
> gastropod_seedsize<-glmer(swallowbind~seedsize+(1|slug_ID),
data=dataset1, family=binomial)

> dispersion_glmer(gastropod_seedsize)
```

```
[1] 1.069778

> summary(gastropod_seedsize)

Generalized linear mixed model fit by the Laplace approximation
Formula: swallowbind ~ seedsize + (1 | slug_ID)
Data: dataset1
AIC BIC logLik deviance
929.3 941.7 -461.7 923.3
Random effects:
Groups Name Variance Std.Dev.
slug_ID (Intercept) 12.128 3.4825
Number of obs: 461, groups: slug_ID, 151
Fixed effects:
Estimate Std. Error z value Pr(>|z|)
(Intercept) -1.304830 0.340498 -3.832 0.000127 ***
seedsize -0.074049 0.009057 -8.176 2.94e-16 ***
---
Signif. codes: 0 ‘***’ 0.001 ‘**’ 0.01 ‘*’ 0.05 ‘.’ 0.1 ‘ ’ 1
Correlation of Fixed Effects:
(Intr)
seedsize -0.403
```
